# Functional Studies on the *LiAG1* Gene of *Lilium* ‘Ice Pink Queen’ in Flower Development

**DOI:** 10.3390/plants14030323

**Published:** 2025-01-22

**Authors:** Lili Xue, Jingqi Dai, Ruyu Fu, Nana Wu, Jiaxuan Yu, Jie Dong, Tao Yang, Jinping Fan

**Affiliations:** College of Horticulture and Landscape, Northeast Agricultural University, Harbin 150030, China; xuelili6277@163.com (L.X.); shuraojing@163.com (J.D.); 15843555033@163.com (R.F.); chenmi0201@foxmail.com (N.W.); 13045406658@163.com (J.Y.); jiedong@neau.edu.cn (J.D.); yangtao@neau.edu.cn (T.Y.)

**Keywords:** *LiAG1*, MADS-box, lily, flower development, male sterile

## Abstract

Lily (*Lilium Asiatica Hybrida*) is a globally known perennial herbaceous bulbous flower, popular for its large, colourful flowers and high economic and ornamental value. However, pollen generation is a severe issue that reduces the cosmetic value of lilies. In this study, the MADS transcription factor LiAG1 was isolated and identified from the *Lilium Asiatica Hybrida* ‘Ice Pink Queen’, a male-sterile variety obtained through several years of hybridisation in our laboratory. qRT-PCR revealed that *LiAG1* expression was greater in lily anthers, especially during the half-opening stage. The transient expression in tobacco demonstrated that LiAG1 was located in the nucleus. In the ‘Ice Pink Queen’ lily, suppression of *LiAG1* using TRV-VIGS (tobacco-rattle-virus-mediated virus-induced gene silencing) resulted in the disappearance of most of the tapetum layer and the absence of the microsporangia. Overexpression of *LiAG1* in transgenic Arabidopsis and tobacco resulted in narrower and more involute leaves, plant dwarfing, earlier blooming, and better pollen viability. Overall, our results suggested that *LiAG1* might play an important role in flower development, especially anther development, of *Lilium Asiatica Hybrida* ‘Ice Pink Queen’.

## 1. Introduction

Flowers are angiosperms’ reproductive organs, and their development is critical for ornamental plant reproduction [1]. Flower organ formation is a complicated process involving several elements that impact and interact with one another. The MADS-box gene family is one of the biggest families of transcription factors in plants [2] and has the ability to affect the whole development process, making it one of the most extensively researched gene families. Plant MADS-box genes are classified into two categories based on their molecular biological characteristics: type I and type II. Type I genes have a shorter length and a simpler structure, consisting simply of the MADS domain. Type II genes exhibit higher complexity and have been extensively studied [3]. The MADS-box type II gene is also known as the MIKC-type gene. The MIKC-type protein structure consists of the MADS domain (M), the intermediate domain (I), the K domain (K), and the C-terminal domain (C) [4]. Depending on the I and K domains, MIKC MADS-box proteins can be further subdivided into two types: MIKCC and MIKC* proteins [5]. So far, most of the well-known MADS genes, such as the ABCDE model of flower development, are basically composed of type II MADS-box genes [6,7]. MADS transcription factors play a key regulatory role in flower development [8,9].

The *AGAMOUS* (*AG*) gene is a type-C gene in the ABCDE model, belonging to a subfamily of the MADS-box gene family. The transcription factor encoded by AGAMOUS belongs to a class of MADS-domain-family proteins. The gene sequence can be divided into five regions from the 5′ end to the 3′ end: N-terminal, M region (MADS-box region encoding about 57 amino acids), I region (Intervening region), K region, and C terminal. Of these, the M region is highly conserved, encoding the MADS protein domain, which can bind target DNA, while regions I and K are relatively conserved and can regulate protein–protein interactions, and they are structural characteristic sequences of transcription factors. The C-terminal is more specific, with two relatively conserved AGⅠ and AGⅡ regions [10,11,12]. The MADS-box gene of class C controls the development of the three-wheeled flower organs of stamen, pistil, and ovule, while only the two-wheeled structure of stamen and pistil is controlled in some plants [13]. Studies have shown that *AG*, as a class C gene in the MIKCC MADS-box family, not only controls the differentiation and development of the stamen and carpel but also regulates the termination of the flower meristem [14]. The *AG* gene has been cloned and analysed in many species, for example, *Arabidopsis thaliana* [15], *Tricyrtis macranthopsis* [16], *Prunus lannesiana* [17], *Amaryllis* [18], etc. Loss of the *AG* gene in *Arabidopsis* leads to changes in the organs in the third and fourth rounds. The petals develop in the place of the stamen, while new flowers are produced in the place of the original pistil and the meristem remains highly active and repeats the process [19]. In addition, some MADS-box genes are important regulators of plant flowering timing. For example, FRUITFULL (FUL), SUPPRESSOR OF OVEREXPRESSION OF CONSTANS1 (SOC1), FLOWERING LOCUS T (FT), and other homologous genes can be influenced by growth stage and environmental factors to promote or inhibit the flowering transformation of plants so as to enhance the adaptability of plants to environmental factors [20]. The *SOC1* gene is an important member of the MADS-box transcription factor family and belongs to the MIKC protein, encoding MADS (M), Intervening (I), Keratin-like (K), and C-terminal (K) protein domains [21]. *Arabidopsis SOC1* controls flowering time and floral organ development [22]. The *FT* gene is a flowering integration factor, and *CONSTANS* (*CO*) regulates flowering by binding to the promoter region of *FT*. In turn, *FT* has a positive effect on *SOC1* [23]. Further activation of the transcription of the *FT* gene promotes the expression of downstream flowering-determining genes [19,24], promotes the up-regulation of *SOC1* gene expression, and initiates the process of plant flowering [25]. The *FUL* gene is a floral allogeneic gene in *Arabidopsis thaliana*, which is highly similar to *APETALA1* (*AP1*), and the negative regulation of *FUL* by *AP1* not only occurs in the young flower primordium stage but also inhibits the expression of *FUL* in sepals and petals in the later development of floral organs [26,27].

At present, the lily is one of the five cut flowers in the world and is loved by people because of its large flowers, bright colours, and beautiful meanings. However, severe pollen contamination has had an impact on its application in the market. Causing male sterility in the lily may alleviate the situation. The new *Lilium Asiatica Hybrida* ‘Ice Pink Queen’ was bred by crossbreeding in our laboratory. Its stamen was not fully developed, its anther was malformed, and no pollen appeared. The malformation of stamens is also one of the manifestations of male sterility, which has advantages in the crossbreeding of pollen-free lilies [28]. Here, a MADS-box member (*LiAG1*) was isolated and identified from the *Lilium Asiatica Hybrida* ‘Ice Pink Queen’. LiAG1 is located in the nucleus, and *LiAG1* is highly expressed in the anthers in the lilium half-blooming stage. Silencing of *LiAG1* in normally developing lilies results in abnormal sporangia development, and overexpression of *LiAG1* in *Arabidopsis thaliana* can promote early flowering of *Arabidopsis*, shorter stigma, and delayed plant growth. Overexpression of *LiAG1* in tobacco was consistent with the phenotype observed in overexpressed *Arabidopsis* plants, and tobacco pollen viability was reduced. These results indicated that *LiAG1* plays a certain role in flower development. This study lays a foundation for further analysis of the role of MADS-box gene in the flower development and male sterility of lily and also provides a theoretical basis for breeding more excellent varieties without anthers and pollens by molecular biological means.

## 2. Results

### 2.1. Morphological Characteristics of Lilium Asiatica Hybrida ‘Ice Pink Queen’ and ‘Elite’

**Figure 1:**
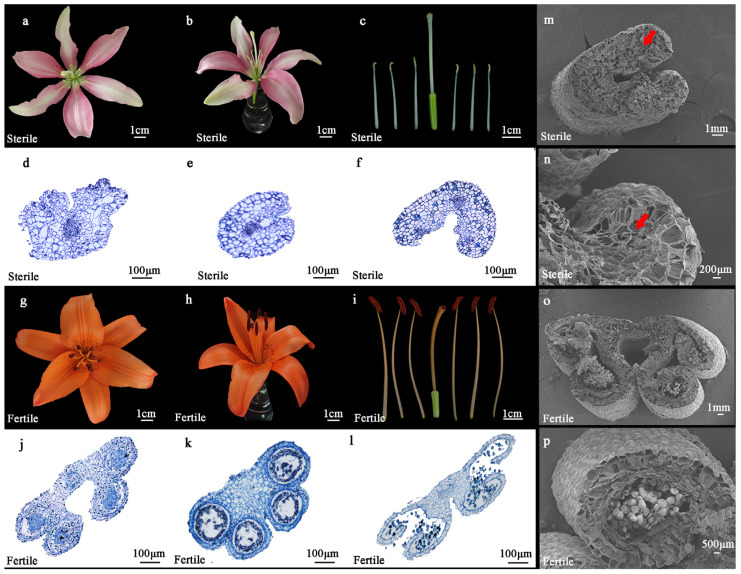
Flower organ morphological and structural characteristics of sterile *Lilium Asiatica Hybrida* ‘Ice Pink Queen’ and fertile ‘Elite’. (**a**–**c**) Flower structure of sterile line. (**g**–**i**) Flower structure of fertile line. (**d**–**f**) Anther transverse microscope observation of sterile lines. (**j**–**l**) Anther transverse microscope observation of fertile lines. (**m**–**p**) Scanning electron microscope observation of anther (**m**,**o**) and microsporangia (**n**,**p**) in sterile and fertile lines. The red arrow points to the sterile defective microsporangia.

Direct observation of the floral organ morphology of the sterile line ‘Ice Pink Queen’ and the fertile line ‘Elite’ revealed that in comparison to ‘Elite’, the petals of ‘Ice Pink Queen’ were flatter and slightly curled inward, the stamens were markedly shorter than the stigmas, and the anther development was incomplete, with no pollen observed on the anther surface during the full blooming stage (Figure 1a–c). In contrast, the petals of ‘Elite’ were rolled outward, exhibiting a more pronounced overall rotation, the lengths of the stamens and stigmas were nearly identical, and the anther surface was densely covered with pollen at the full blooming stage (Figure 1g–i).

The paraffin section showed that with the development of anthers, the ‘Elite’ anther cells developed normally, forming four microsporangia; the tapetum developed normally, forming pollen granulocytes normally; and the pollen was normally dispersed with the rupture of the microsporangia (Figure 1j–l). With the development of anthers, the ‘Ice Pink Queen’ could not form four microsporangia, and the cells in the centre of the inner wall were clustered and narrowed, no tapetum cells were found, and no pollen cells were formed (Figure 1d–f).

Scanning electron microscopy was employed to examine the anther structure of both fertile and sterile lines. In the fertile line ‘Elite’, the anthers developed normally, featuring four well-formed microsporangia containing fully developed pollen grains (Figure 1o,p). Conversely, the anthers of the sterile line ‘Ice Pink Queen’ failed to develop four distinct microsporangia and predominantly exhibited hollow and fragmented cellular structures incapable of producing viable pollen grains (Figure 1m,n). These findings are corroborated by observations made through paraffin sectioning. Collectively, these results suggest that the anther development of the *Lilium Asiatica Hybrida* ‘Ice Pink Queen’ is defective, leading to the inability to form functional pollen grains during anther maturation, potentially resulting in male sterility.

### 2.2. Transcriptome Data Analysis of Flower Buds in the ‘Ice Pink Queen’ and ’Elite’

The DNA Nanoball sequencers system (DNBSEQ platform) was employed for the transcriptome sequencing of lily flower buds. The original data set comprised 38.66 Gb, and 148,193 Unigene sequences were obtained following assembly and redundancy removal. To guarantee the quality of the data for analysis, the data were filtered, and the proportion of clean reads was above 97.6%. The clean reads were assembled using Trinity, the quality indicators of the Unigene after clustering were calculated (Table S1), and their length distribution was plotted (Figure S1). The candidate coding regions in Unigene were identified using TransDecoder software (https://transdecoder.github.io (accessed on 10 June 2023)), and the predicted CDS length distribution was compared by BLAST (Figure S2).

**Figure 2:**
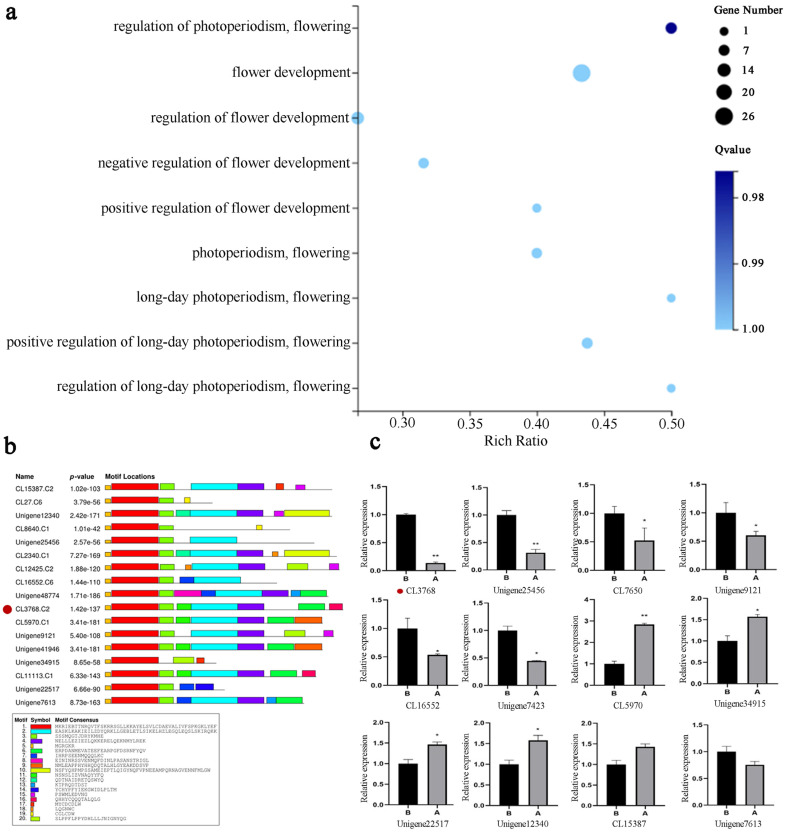
Transcriptome data analysis. (**a**) Differential gene GO enrichment bubble map. (**b**) Comparison of conserved motifs of MADS-box differential proteins. (**c**) Expression of *MADS-box* genes in transcriptome data. A: *Lilium Asiatica Hybrida* ‘Ice Pink Queen’. B: *Lilium Asiatica Hybrida* ‘Elite’ is the control group. All data were derived from at least three biological replicates and are expressed as the mean ± standard deviation; * indicates a significant difference between A and B (*p* < 0.05), ** indicates a significant difference between A and B (*p* < 0.01).

In order to identify key genes, differentially expressed genes were identified in sterile and fertile lines. A total of 48,485 significantly differentially expressed genes were identified, comprising 17,311 genes that were up-regulated and 31,174 genes that were down-regulated (Figure S3). *p* values were adjusted using the Benjamini–Hochberg method. Differentially expressed genes were detected using padj < 0.05 and |log2 Fold change| > 1. A Gene Ontology (GO) enrichment analysis was conducted on the transcriptome data (Figure 2a), and a GO Term analysis was performed throughout the process of flower development [29]. This analysis identified 22 unique genes. Subsequently, an additional screening process was undertaken on the 22 genes identified through NR annotation (Table 1). The remaining genes were then subjected to further analysis, with those that were unannotated or unrelated to anther development being excluded. This process led to the selection of the CL3768 gene as the target gene for further study.

An analysis of the selected CL3768 gene in the transcriptomes revealed that it exhibited conserved domains and motifs characteristic of the MADS-box gene family (Figure 2b). These findings suggest that the gene belongs to class C genes in the MADS-box family, displaying strong C-terminal specificity and two relatively conserved AGⅠ and AGⅡ regions. This sequence is characteristic of the *AG* gene [30], and thus CL3768 has been designated *LiAG1*. A heat map analysis of *MADS-box* genes in the transcriptome revealed notable discrepancies in the expression of target genes (Figure S4). Twelve *MADS-box* genes were selected according to the transcriptome, and the transcriptome was verified by quantitative reverse transcription polymerase chain reaction (qRT-PCR). The results of the qRT-PCR demonstrated a significant difference in the expression of the *LiAG1* gene (Figure 2c), suggesting that this gene may play a pivotal role in anther development. The expression trends of the other genes were consistent with those observed in the transcriptome, thereby confirming the reliability of the transcriptome data.

### 2.3. LiAG1 Is a MADS-Box Member in Lily and Is a Nuclear Localisation Protein

**Figure 3:**
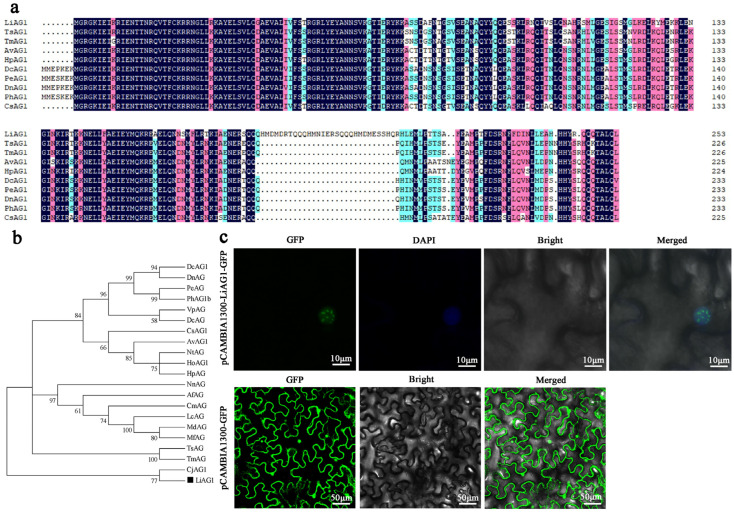
Comparison of cDNA sequence and amino acid sequence, phylogenetic tree, subcellular localisation analysis of LiAG1 of ‘Ice Pink Queen’. (**a**) DNAMAN 9.0 was used to compare the amino acid sequence derived from AG1 protein. AG1 protein sequences from other species are as follows: included TsAG1 (BAU88524.1) in *Tricyrtis* sp. Shinonome, TmAG1 (BBE10899.1) in *Tricyrtis macranthopsis,* AvAG1 (BAD18011.1) in *Asparagus virgatus*, HpAG1 (ACB70410.1) in *Hosta plantaginea,* DcAG1 (AAZ95250.1) in *Dendrobium crumenatum*, PeAG1 (XP_020582512.1) in *Phalaenopsis equestris,* DnAG1 (KAI0492124.1) in *Dendrobium nobile*, PhAG1 (ARX76300.1) in *Phalaenopsis hybrid cultivar,* CsAG1 (AAS67610.1) in *Crocus sativus*. The black, red, and blue parts indicate homology = 100%, ≥75%, ≥50%, respectively. (**b**) Phylogenetic tree of AG1 protein using adjacency method and 1000 bootstrap repeats. Accession numbers of the amino acid sequences obtained from NCBI are listed below: TsAG1 (BAU88524.1) in *Tricyrtis* sp. Shinonome, TmAG1 (BBE10899.1) in *Tricyrtis macranthopsis,* AvAG1 (BAD18011.1) in *Asparagus virgatus*, HpAG1 (ACB70410.1) in *Hosta plantaginea,* DcAG1 (AAZ95250.1) in *Dendrobium crumenatum*, PeAG1 (XP_020582512.1) in *Phalaenopsis equestris,* DnAG1 (KAI0492124.1) in *Dendrobium nobile*, PhAG1 (ARX76300.1) in *Phalaenopsis hybrid cultivar,* CsAG1 (AAS67610.1) in *Crocus sativus,* VpAG1 (KAG0465477.1) in *Vanilla planifolia*, NtAG1 (ABQ28694.1) in *Narcissus tazetta subsp. Chinensis,* HoAG1 (AAD19360.2) in *Hyacinthus orientalis,* NnAG1 (XP_010272685.1) in *Nelumbo nucifera,* AfAG1 (ALV83435.1) in *Aristolochia fimbriata,* CmAG1 (RWR89169.1) in *Cinnamomum micranthum,* LcAG1 (AFH74375.1) in *Liriodendron chinense,* MdAG1 (AFH74369.1) in *Manglietia duclouxii,* MfAG1 (AFH74385.1) in *Magnolia figo,* CjAG1 (ASY97759.1) in *Cercidiphyllum japonicum.* (**c**) Subcellular localisation of LiAG1. The Fusion proteins pCAMBIA1300::LiAG1-GFP and pCAMBIA1300::GFP as a control protein were detected with a confocal laser scanning microscope.

The full length of the ORF region of the *LiAG1* gene is 765 bp, encoding 254 amino acids in total (Figure 3a). The amino acid sequence comparison between *LiAG1* and homologous proteins of other species indicates that the LiAG1 protein has a conserved domain common in the MADS-box family. The selected plant AG1 protein has two relatively conserved AGⅠ and AGⅡ regions in the C terminal. The amino acid sequence of *LiAG1* was analysed by multiple sequence alignment using the neighbourhood linkage method (NJ), and a phylogenetic tree was constructed. The results showed that *LiAG1* and *CjAG1* were highly homologous (Figure 3b).

To determine the subcellular localisation of LiAG1, a pCAMBIA1300::LiAG1-GFP overexpression vector was constructed for subcellular localisation detection. Compared to the empty carrier, the green fluorescence signal of the pCAMBIA1300::LiAG1-GFP fluorescent carrier appeared only in the nucleus (calibrated by DAPI staining) (Figure 3c). The results of subcellular localisation indicate that LiAG1 is a nuclear localisation protein.

### 2.4. LiAG1 Gene Is Mainly Expressed in Anthers

**Figure 4:**
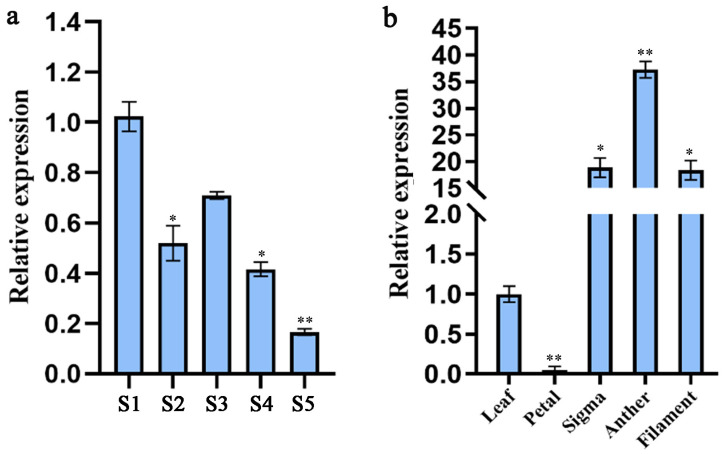
*LiAG1* gene expression analysis. (**a**) Expression analysis of *LiAG1* at different periods of ‘Ice Pink Queen’. S1: green-bud stage; S2: pink-bud stage; S3: half-open period; S4: blooming period; S5: decline period. S1 is the control group. (**b**) Tissue-specific expression analysis of *LiAG1* gene. Leaf is the control group. All data were derived from at least three biological replicates and are expressed as the mean ± standard deviation; * indicates *p* < 0.05, ** indicates *p* < 0.01.

Real-time PCR was used to analyse the relative expression levels of the *LiAG1* gene in different tissues and at different flowering stages of the Asiatic lily ‘Ice Pink Queen’ (Figure 4a,b). The expression level of the *LiAG1* gene was significantly different at different flowering stages. The general trend was to decrease, and it would increase at the half-blooming stage. The expression level of the *LiAG1* gene reached the lowest level at the decay stage, about 0.16 times of that at the green-bud stage. There were also significant differences in the amount of expression between different tissues, with very high expression in anthers and almost no expression in petals. These results indicated that the lower the expression of *LiAG1* gene, the more it affected anther formation, and it mainly promoted the formation of stamens from the blooming stage to the decay stage. the *LiAG1* gene was mainly expressed in anthers, and the anthers of ‘Ice Pink Queen’ lily are incomplete and do not produce pollen, so it is speculated that *LiAG1* may play an important role in the process of anther development and male sterility of ‘Ice Pink Queen’ lily. Taken together, these observations support the potential function of *LiAG1* as a class C gene in flower organ development.

### 2.5. Silenced LiAG1 Affects Anther Development

**Figure 5:**
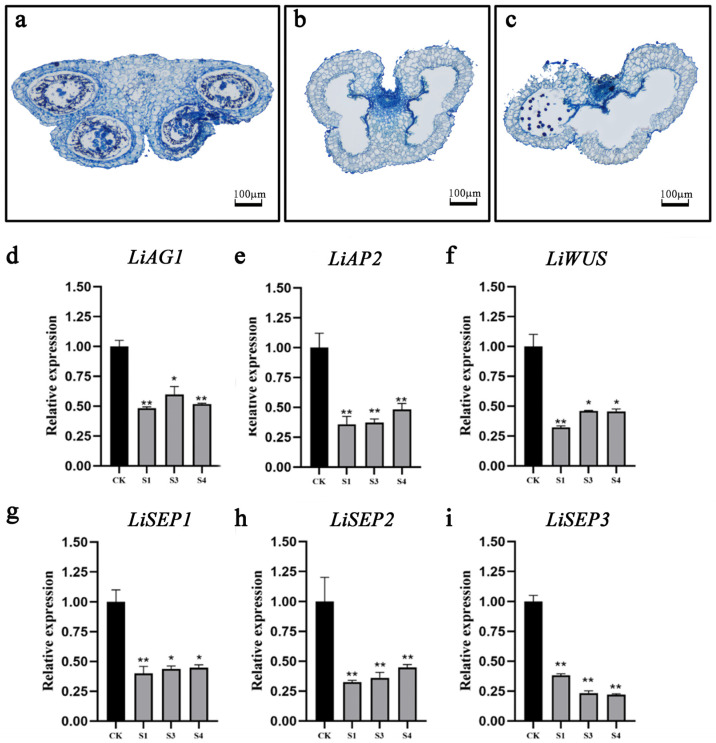
Silencing of LiAG1 in lily anthers by VIGS. (**a**–**c**) Transverse morphologies of anthers in TRV2 and TRV2-LiAG1 lilies. Three independent experiments were performed, and two representative pictures (**b**,**c**) are shown. (**d**–**i**) The expression patterns of key flowering genes in positive silent plant. CK is the control group. All data were derived from at least three biological replicates and are expressed as the mean ± standard deviation; * indicates a significant difference between the experimental group and CK (*p* < 0.05); ** indicates a significant difference between the experimental group and CK (*p* < 0.01).

In order to ascertain whether *LiAG1* affects the anther development of the lily, a VIGS analysis was conducted using the ‘Elite’ lily. Anthers exhibiting a superior silencing effect were selected for paraffin sectioning. In comparison to the TRV control, the lily anthers that had been subjected to silencing were situated in close proximity to the septum. Additionally, the wall cells of the microsporangia exhibited a reduction in size and clustering, which impeded their ability to expand normally. The majority of the tapetum layer underwent a process of disappearance, with only a small proportion remaining intact (Figure 5a). The chamber remained intact, and no pollen was produced (Figure 5b). The anthers were absent, with only three anthers formed and a minimal quantity of pollen present in one of them (Figure 5c).

To determine whether silencing of *LiAG1* affects the expression of genes involved in flower development, the expression levels of these genes were analysed and detected. The analysis of the expression levels of flower development genes in three lily petals exhibiting silencing demonstrated that the silencing level of the *LiAG1* gene in the petals reached approximately 50% (Figure 5d), which was consistent with the systematic silencing effect of TRV. The *LiAP2* genes are classified as class A genes, which inhibit *LiAG1* gene expression. The *LiSEP1*, *LiSEP2*, and *LiSEP3* genes are classified as class E genes, which cooperate to complete the development of flower organs. In the *AGAMOUS*(*AG*)-*WUSCHEL(WUS)* feedback pathway, *WUS* activates *AG* expression in the stamen and carpel proembryo, thereby initiating reproductive development and antagonising *WUS* activity [31]. A reduction in the expression level of the *LiAG1* gene resulted in a corresponding decrease in the expression levels of *WUS*, *SEP1*, *SEP2*, and *SEP3*, which are synergistic with stamen development, and *AP2*, which inhibits *AG* and affects anther development (Figure 5e–i).

### 2.6. Overexpression of LiAG1 Can Promote Early Flowering and Stigma Shortening of Arabidopsis

**Figure 6:**
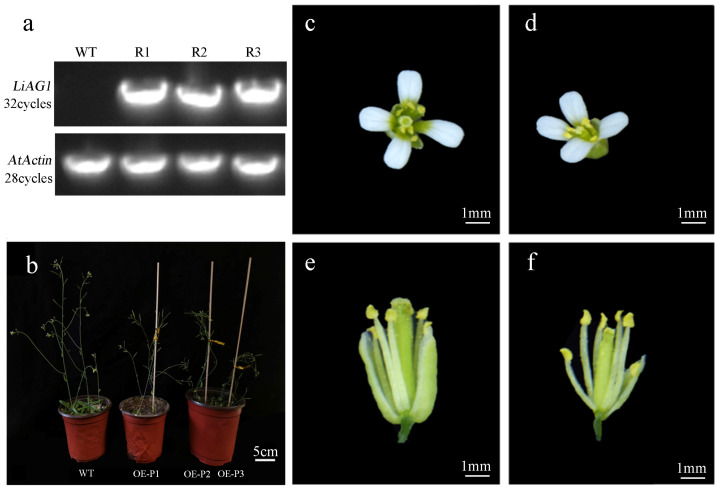
Growth status, flowering time, and flower phenotype of transgenic *Arabidopsis*. (**a**) Transgenic *Arabidopsis* was identified by SqRT-PCR and agarose electrophoresis. WT: wild-type control; R1–R4: T2-generation *Arabidopsis*. (**b**) Growth state of transgenic *Arabidopsis*. WT: control group. (**c**–**f**) Comparison of flower organs between transgenic T3-generation *Arabidopsis* and wild-type *Arabidopsis*. (**c**,**e**) Wild *Arabidopsis*. (**d**,**f**) Transgenic *Arabidopsis* OE-P1 strain.

Three transgenic lines (OE-R1, OE-R2, OE-R3) were performed by semiquantitative PCR. Using *β-ACTIN* (*AtACTIN*) as a reference gene, the expression of *LiAG1* in transgenic lines was analysed. There was no amplification to bands from wild-type plants but amplification to bands from all transgenic plants, and these results indicated that the *LiAG1* gene was correctly transcribed (Figure 6a). Phenotypic analysis showed that the flowering time of all *LiAG1* transgenic *Arabidopsis* plants was about 5 days earlier than that of wild *Arabidopsis* plants. In addition, the overall growth of the plant was weak, the leaves were narrow and curled at the front (Figure 6b), the petals were closely arranged, and the stigma was thinner and shorter than that of the stamen (Figure 6c–f).

### 2.7. Expression of Key Flowering-Related Genes of LiAG1 Transgenic Arabidopsis thaliana

**Figure 7:**
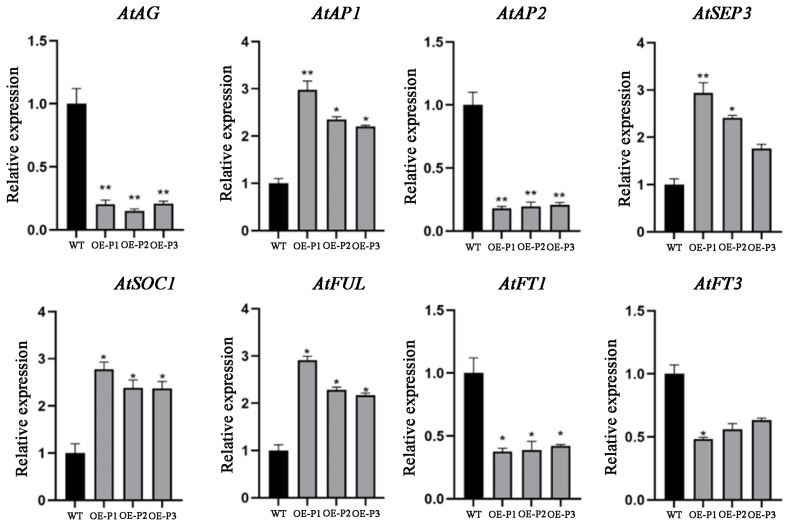
Flower development gene expression analysis of transgenic *Arabidopsis thaliana*. WT: wild-type *Arabidopsis Thaliana* strain; OE-P1, OE-P2, OE-P3: transgenic *Arabidopsis* lines. All data were derived from at least three biological replicates and are expressed as the mean ± standard deviation. * indicates a significant difference between the transgenic lines and WT (*p* < 0.05), ** indicates a significant difference between the transgenic lines and WT (*p* < 0.01).

The *AtAP1*, *AtSOC1*, *AtFT1*, *AtFT3*, *AtAP2*, *AtFUL*, and *AtSEP3* genes have been identified as playing a significant role in the regulation of *Arabidopsis thaliana*. To gain further insight into the function of the *LiAG1* gene, we examined the expression levels of key flowering genes in *Arabidopsis thaliana* with *LiAG1* overexpression. In comparison to the wild type, the *AtAP1*, *AtSOC1*, *AtFUL*, and *AtSEP3* genes, which are associated with early flowering and floral organ differentiation, exhibited a notable increase in expression levels in the overexpressed strains. The inhibitory flowering genes *AtFT1*, *AtAP2*, and *AtFT3* were significantly down-regulated (Figure 7), indicating that the overexpression of *LiAG1* promoted the expression of early flowering genes, advanced the flowering stage, and affected the development of flower organs.

### 2.8. Overexpression of LiAG1 Can Promote Early Flowering and Stigma Shortening in Tobacco

**Figure 8:**
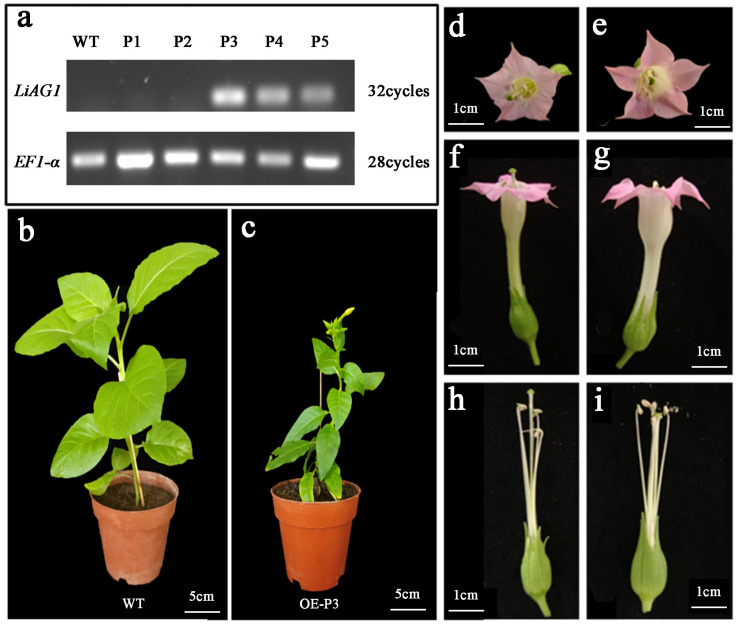
Growth status, flowering time, and flower phenotype of transgenic tobacco. (**a**) Transgenic tobacco was identified by qRT-PCR and agarose electrophoresis. WT: wild-type control; P1–P5: T0-generation transgenic tobacco plants to be tested. (**b**) Wild-type tobacco growth status. (**c**) Transfer *LiAG1* gene tobacco growth status. (**d**,**f**,**h**) Observation of wild tobacco flower phenotype. (**e**,**g**,**i**) Observation of tobacco with *LiAG1* gene flower phenotype.

To further verify the function of the *LiAG1* gene, tobacco leaf discs were selected for stable genetic transformation of the *LiAG1* gene. Five T0-generation seedlings were successfully grown, and semiquantitative PCR identification showed that P3 had strong overexpression of *LiAG1* (Figure 8a). Therefore, P3 was selected for further analysis. We found that the overexpressed strain P3 had smaller and narrower leaves, significantly reduced leaf area, thinner stems, and shorter plants compared to the wild type (Figure 8b,c) and flowered about 20 days earlier. Phenotypic observation of the floral organs of transgenic tobacco showed that compared to the wild type, the floral bobbin of transgenic plants was thicker, and the stigma was shorter than the stamen (Figure 8d–i). This is consistent with the phenotype observed in overexpressed *Arabidopsis* plants.

### 2.9. Overexpression of LiAG1 Decreased the Viability of Tobacco Pollen

**Figure 9:**
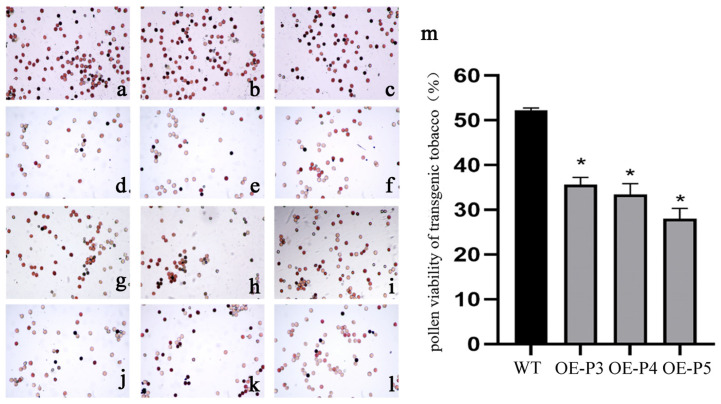
Comparison of pollen viability between transgenic tobacco and wild-type tobacco. (**a**–**c**) Wild-type tobacco. (**d**–**l**) Three strains of transgenic tobacco with *LiAG1* gene, with three pictures in each row from left to right representing one strain. (**m**) Bar chart of pollen viability in transgenic tobacco and wild-type tobacco, WT: wild-type tobacco; OE-P3, OE-P4, OE-P5: transgenic tobacco strains with *LiAG1* gene. All data were derived from at least three biological replicates and are expressed as the mean ± standard deviation. * indicates *p* < 0.05.

We tested the pollen viability of three *LiAG1*-overexpressing tobaccos and one wild-type tobacco. Compared with the wild-type tobacco, TTC staining showed that the pollen quantity of the three transgenic tobaccos was significantly lower than that of the wild-type tobacco, and the pollen viability was significantly reduced (Figure 9a–l). The pollen activity of transgenic tobacco was about 0.6 times that of wild-type tobacco pollen (Figure 9m). In summary, we speculated that overexpression of the *LiAG1* gene would affect the viability of tobacco pollen, reduce it, and affect fertility.

### 2.10. Expression of Key Flowering-Related Genes in LiAG1 Transgenic Tobacco

**Figure 10:**
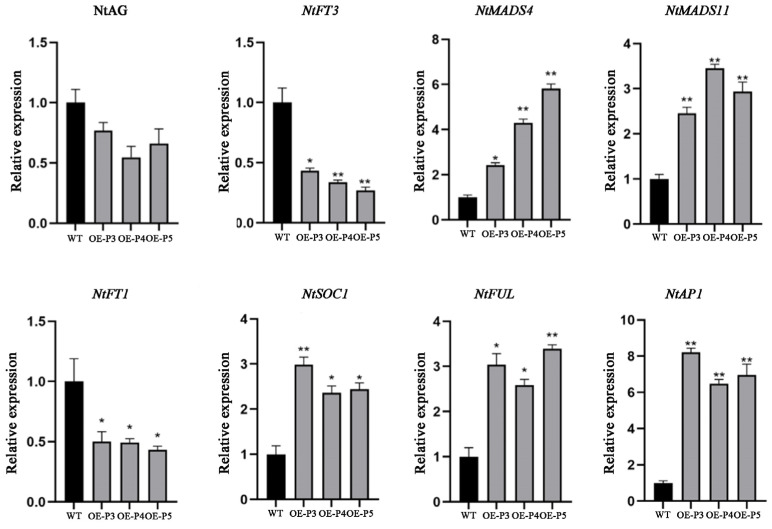
Analysis of gene expression in transgenic tobacco flower development. WT: wild-type tobacco; OE-P3, OE-P4, OE-P5: tobacco lines with *LiAG1* gene. All data were derived from at least three biological replicates and are expressed as the mean ± standard deviation; * indicates a significant difference between the transgenic lines and WT (*p* < 0.05), and ** indicates a significant difference between the transgenic lines and WT (*p* < 0.01).

To investigate the relationship between *LiAG1* and floral-organ-related genes, we selected three overexpressed strains OE-P3, OE-P4, OE-P5 and wild-type tobacco. The expression levels of genes related to flower development, such as *NtAG*, *NtMADS4*, *NtMADS11*, *NtFT1*, *NtFT3*, *NtSOC1*, *NtAP1*, and *NtFUL*, are detected in the three overexpressed lines, and the expression levels of *NtAG* are significantly reduced, with a more pronounced decrease in OE-P4. Compared with the wild type, the expression levels of *NtMADS4*, *NtMADS11*, *NtSOC1*, and *NtAP1* genes related to early flowering are significantly up-regulated in the overexpressed lines, and the overexpression of *NtMADS4* and *NtMADS11* genes can also cause plant dwarfing. The expression levels of *NtFT1* and *NtFT3*, which inhibit flowering genes, decreased significantly (Figure 10), indicating that overexpression of *LiAG1* promoted the expression of early flowering genes, advanced the flowering stage, and led to plant dwarfing.

## 3. Discussion

In this study, we conducted a comparative analysis of the overall flower organ morphology of the sterile Asiatic lily line ‘Ice Pink Queen’ and its fertile line ‘Elite’ (Figure 1a–c,g–i). Furthermore, we employed cytological observation to investigate the differences in anther development between the two lines. The results demonstrated that in comparison with the fertile line of the Asiatic lily ‘Elite’, the sterile line of the Asiatic lily ‘Ice Pink Queen’ was unable to form the structure of four drug chambers as the drug chamber developed. The cells situated in the centre of the inner wall were clustered and narrowed, no tapetum cells were identified, and pollen cells could not be formed (Figure 1d–f,m,n). It is therefore evident that a detailed examination of the sterile line of the Asiatic lily ‘Ice Pink Queen’ will prove invaluable in enhancing the ornamental quality of this species.

In order to gain further insight into the male sterility mechanism of the ‘Ice Pink Queen’ lily, transcriptome sequencing technology was employed to analyse the transcriptome data of flower buds of two species of Asian lily, with the objective of deepening our understanding of the expression changes of differential genes. A total of 17,311 differential genes were found to be up-regulated, while 31,174 were down-regulated (Figure S3). Following the GO Term analysis of the transcriptome throughout flower development, NR annotation was employed to exclude unannotated genes and genes unrelated to anther development. Ultimately, *AG1* (CL3768) was identified as the target gene for further investigation. Further analysis demonstrated that the expression level of the *LiAG1* gene in the sterile line of the Asiatic lily ‘Ice Pink Queen’ was lower than that observed in the fertile line. This led to the hypothesis that the expression level of the *LiAG1* gene may influence anther development and pollen phenotype.

*AG*, as a class C transcription factor, has been demonstrated to regulate the formation of female organs and stamens in a range of plant species [32]. The *AG* homologous gene *ThtAG1* has been demonstrated to influence floral organ characteristics and determine tissue specificity [33]. The overexpression of the *AG* homologous gene *TaAG* in wheat has been observed to result in significantly advanced reproductive differentiation and the formation of abnormal stamens and stigmas in *Arabidopsis thaliana* [34]. The overexpression of the Jatropha *AG* homologous gene, *JcAG*, in Arabidopsis has been observed to result in early flowering, delayed development of the anther and stamen, and pistillation of sepals [35]. The *Arabidopsis* SPOROCYTELESS (SPL)/NOZZLE (NZZ) gene plays a crucial role in the early stages of anther cell division and differentiation, both of which are essential for sporogenesis. In the *SPL*/*NZZ* mutant anthers, the formation of sporogenous cells appears normal; however, subsequent cell division is defective, resulting in the loss of sporogenous cells and non-reproductive tissues, including the tapetum layer [36,37,38]. It has been demonstrated that the *AG* gene enhances the expression of the SPL/NZZ gene and is essential for sporogenesis [39]. This indicates that *AG* plays a role in regulating male fertility, at least in part, through the mediation of *SPL/NZZ*. Furthermore, the *AG* protein may act as a direct regulator of *SPL* expression [40]. In our study, the *LiAG1*-silencing line of the ‘Elite’ lily exhibited a reduction in and aggregation of anther cells in the inner wall of the septum, which impeded their normal development and resulted in the disappearance of the majority of tapetum cells. It was postulated that the *LiAG1* gene may exert an influence on the development of cells comprising the drug compartment and tapetum. In conjunction with the ABCDE model, the qPCR data indicated that a reduction in the expression level of the *LiAG1* gene resulted in a decline in the expression levels of the *SEP1*, *SEP2*, and *SEP3 E* genes, which exerted an A-mediated synergistic effect on anther development; the *AP2* A gene, which inhibited its activity; and the *WUS* gene, which maintained *AG1* activity. Such alterations may impact anther development, potentially resulting in the deformation of other floral organs.

Overexpression of *LiAG1* in tobacco and *Arabidopsis* showed a decrease in the expression of their own endogenous *AG* gene in both plants, which qPCR data showed. The expression levels of *MADS-box* genes associated with early flowering were significantly increased, while the expression levels of *FT* genes that inhibit flowering were significantly decreased (Figure 7 and Figure 10). Therefore, it was speculated that overexpression of the *LiAG1* gene could achieve the effect of early flowering by regulating the expression of early flowering genes. Phenotypic analysis showed that *LiAG1* could dwarf the plants, narrow the leaves, shorten the stigma, and advance the flowering time. *LiAG1* is a homologous gene of *AtAG*. Previous researchers found that overexpressing the *AG* gene from other plants in *Arabidopsis thaliana* resulted in transgenic Arabidopsis with weaker growth potential, dwarfed plants, narrowed leaves with curled front ends, and some yellowing leaves, for example, the *Tageteserecta TeAG1* gene [41] and Aechmea fasciata *AfAG* gene [42]. Our results are also consistent with this point. In *Arabidopsis thaliana*, previous studies found that the MADS transcription factor family members encoded by the plant C-type floral organ identity gene *AG* play a central regulatory role in the termination regulation of floral meristem development and the determination of floral organs in stamens and pistils [15,43]. Studies have shown that overexpression of *MtAGa* and *MtAGb* in alfalfa will result in petal stamenisation [44]. However, no obvious phenotypic changes in floral organs were observed in the transgenic plants, which may be due to the non-compatibility of the heterologous transformation species, i.e., lily is a monocotyledonous plant, whereas Arabidopsis thaliana and Nicotiana tabacum are dicotyledonous plants. In conclusion, overexpression of the *LiAG1* gene affects plant height, leaves, and stigmas and has certain regulatory effects on flowering time. However, further verification is needed to determine whether the *LiAG1* gene affects the transformation of lily floral organs.

## 4. Materials and Methods

### 4.1. Plant Materials

All lily plants were grown at the Horticultural Experiment Station of the Northeast Agricultural University. In this study, two lily cultivars were used. The first is the *Lilium Asiatica Hybrida* ‘Ice Pink Queen’, which is unable to produce pollen due to incomplete stamens. *Lilium* ‘lce Pink Queen’ was selected from the cross of ‘Pollyanna’ as female parent and ‘Dark Beauty’ as male parent by conventional crossbreeding method. Both parents were introduced from the Netherlands and preserved and planted in the lily resource nursery of Northeast Agricultural University. The second is the *Lilium Asiatica Hybrida* ‘Elite’, a typical pollen-producing cultivar. The seeds of *Nicotiana tabacum*, *Nicotiana Nicotiana tabacum* (‘95’ Tobacco), and *Arabidopsis* Columbia-0 were stored in the Garden Plant Laboratory of Northeast Agricultural University, and the above plants were planted in the Garden Plant Genetics and Breeding Laboratory of Northeast Agricultural University. The light conditions of plant culture were vegetative growth for 12/12 h and reproductive growth for 14/10 h. The humidity was 40–60%; the temperature was 22–24 °C.

### 4.2. Morphological and Histological Analysis

The whole flower organs of the Asiatic lilies ‘Ice Pink Queen’ and ‘Elite’ were observed morphologically, and the buds of 2 cm, 3 cm, and 6 cm were analysed histologically by paraffin section method [45]. The experiment was observed by DMil microscope (Leica, Weztlar, Germany) and photographed by Hitachi S3400N scanning electron microscope (Hitachi, Tokyo, Japan) [46].

### 4.3. Transcriptome Differential Expression Gene Screening

The flower buds of the ‘Ice Pink Queen’ and ‘Elite’ were each collected with 0.5 g of bulb diameter of about 5 cm. Three replicates were set up, and transcriptome sequencing was commissioned from Beijing Genomics institution (BGI, Shenzhen, China) to obtain transcriptome data. Genes related to flower development were screened from the transcriptome data by GO enrichment analysis. The selected genes related to flower development were verified by qRT-PCR (Table S2).

### 4.4. RNA Extraction and Gene Cloning

Total RNA was extracted from ‘Ice Pink Queen’ lily buds according to a TransZol Plant kit (TransGen Biotech, Beijing, China), and the first-strand cDNA was synthesised using an EasyScript^®^ reverse transcription system (TransGen Biotech, Beijing, China). The first-strand cDNA was synthesised using the EasyScript^®^ reverse transcription system (TransGen Biotech, China). Open reading frames (ORFs) of the *LiAG1* gene were amplified using primers designed based on a transcriptome sequencing screen. For all PCR products, a pEASY^®^-Blunt Zero vector was used with a pEASY^®^-Blunt Zero Cloning Kit (TransGen Biotech, China). The recombinant vector was then transformed into E. coli DH5α, and DNA extracted from these cells was sequenced to identify positive clones. The primer sequences used in this study are listed in Table S2.

### 4.5. Sequence Comparison and Phylogenetic Analysis

DNAMAN 9.0 was used to compare the protein sequence and amino acid sequence of the LiAG1 gene of the ‘Ice Pink Queen’ with the *AG1* gene of other plants. Amino acid sequence homology was analysed using Blast. The phylogenetic analysis of *LiAG1* protein was performed using MEGA-X, the evolutionary tree was constructed using the neighbour-joining method with a test method of 1000 bootstrap repeats, and the mapping software Evolview (www.evolgenius.info/evolview/#/login (accessed on 10 June 2023)) was applied to beautify the protein evolutionary tree.

### 4.6. Subcellular Localisation of LiAG1

The fusion expression vector pCAMBIA1300::LiAG1-GFP was constructed by inserting the CDS sequence of the *LiAG1* gene without stop codon into the pCAMBIA1300::GFP vector using the specific primers egLiAG1-F and egLiAG1-R (Table S2). The sequenced fusion (pCAMBIA1300::*LiAG1*-GFP) was transferred into Agrobacterium GV3101 and used to infect 4-week-old tobacco leaves. The plasmid without LiAG1 (pCAMBIA1300::GFP) was used as a control. The infested tobacco seedlings were incubated at 22 °C and 50% humidity for 1 d under light protection and then incubated normally for 1.5 days. DNA stain 4,6-diaminidine 2-phenyldole DAPI was used for staining calibration, and the DAPI concentration was 1 g/mL. The fluorescence expression of the cells was detected using a laser confocal microscope (Olympus FV3000, Ina-shi, Japan).

### 4.7. Organ-Specific Expression of LiAG1

To verify the specific location of the *LiAG1* gene in lily flowers and the changing trend of expression during flower development, real-time quantitative PCR was performed using a qTOWER3 G real-time PCR instrument (Analytik Jena, Jena, Germany). Petals, filaments, anthers, pistils, and leaves of the lily at five stages of flowering and half-flowering were collected, and cDNA was reverse-transcribed after RNA extraction using the same method as in Section 4.4. The lily *Actin* gene, accession number KJ543466, was selected as the internal reference gene. Quantitative real-time PCR experiments were conducted using the protocols outlined in the instructions for the ChamQ Universal SYBR qPCR Master Mix (Vazyme Biotech Co., Ltd., Nanjing, China), and the Master mixture was prepared on ice. Each 20 μL reaction volume included 10 μL ChamQ Universal SYBR qPCR Master Mix (2×), 0.4 μL forward primer, 0.4 μL reverse primer, 2 μL cDNA solution (as a template), and 7.2 μL sterile water. The reaction protocol was 95 °C, 30 s; 95 °C, 5 s; and the dissolution curves were enhanced by heating from 65 °C to 95 °C at a rate of 0.5 °C/5 s at 58 °C, 30 s, and 40 cycles. Each sample went through three biological and three technical repetitions. The expression levels of each gene were calculated using the 2^−ΔΔCt^ comparison threshold cycle method. After processing the experimental data with SPSS, charting was performed with GraphPad Prism 8.0.

### 4.8. Virus-Induced LiAG1 Gene Silencing and Identification

A 155-bp gene-specific fragment was cloned into the pTRV2 vector using cDNA as a template to obtain pTRV2-*LiAG1*. Primer sequences used to generate the TRV vector are listed in Table S2. The recombinant plasmid was transferred into Agrobacterium tumefaciens GV3101. The bacterial solution was resuspended to OD600 = 1.0, and then the Agrobacterium strain carrying TRV1 was mixed with the Agrobacterium strains carrying TRV2 (negative control) (1:1), while the Agrobacterium strain carrying TRV1 was mixed with the Agrobacterium strain carrying pTRV2-LiAG1 (1:1) and stored at room temperature in the dark for 3 h for standby. A total of 80 green lilies about 2 cm in size were ‘refined’ in advance, and the pedicel length of each was retained at 5 cm and randomly divided into four groups, and the pedicel was submerged into the TRV1:TRV2-*LiAG1*-1 (1:1) mixture at the time of infestation, while for the control group, the pedicel was submerged into the TRV1:TRV2 (1:1) mixture and placed in a vacuum chamber. For the control group, the flower stalks were submerged into the TRV1:TRV2 (1:1) mixture and placed in a vacuum pump at a pressure of 0.07 kPa for 5 min; then, the basal cut surfaces of the flower stalks were washed with sterile water, and the stalks were inserted into bottles containing distilled water for 1 d. Afterwards, they were placed in an artificial climate chamber at a controlled temperature of 20 °C to wait for the flowering of the flowers, during which time their water could be replaced once [47].

After 10 days of silencing, the CTAB crude genomic DNA extraction method [48] and TRV2 universal primers (Table S2) were used for PCR identification to detect whether lily buds were silenced. The positive silent flower buds were sectioned in paraffin to observe anther development, RNA was extracted, and the cDNA template was obtained by reverse transcription. qRT-PCR was used to determine the expression of genes related to flower development after silencing of the ‘essence’ *LiAG1* gene. The specific primers for lily qPCR are shown in Table S2. The LrTIP41 gene (GenBank: KJ543466) was selected as an internal control. Three techniques were repeated for each sample, and the 2^−ΔΔCt^ method was used to calculate the relative gene expression level. The *p*-value of sample difference significance < 0.05 was considered statistically significant. GraphPad Prism 8.0 was used for data processing and visualisation.

### 4.9. Generation of Transgenic Arabidopsis Thaliana Strains and Identification of Positive Plants

The GV3101::*LiAG1* vector was introduced into *Arabidopsis* by means of infection of the inflorescence [49]. T1 and T2 transgenic seeds were collected, and it was observed that all T2 seeds (T3) exhibited transgenic characteristics to a considerable extent [50]. The selected transgenic T3-generation plants were transplanted into the substrate and cultivated under identical conditions (20–22 °C and a 16 h/8 h light cycle). This resulted in the isolation of T3-generation *Arabidopsis thaliana* strains exhibiting overexpression of *LiAG1*.

RNA extracted from the T3 generation of *Arabidopsis thaliana* was reverse-transcribed into cDNA, which served as the template. The T3-generation transgenic *Arabidopsis* Thaliana plants were identified by RT-PCR using the full-length primers LiAG1-F and LiAG1-R specific to the *LiAG1* gene (Table S2). Concurrently, the *AtActin* gene (GeneBank:AT3G18780) was subjected to the design of specific primers (Table S2), which were then employed in conjunction with the *LiAG1* gene-specific primers q-LiAG1-F and q-LiAG1-R (Table S2) in a semiquantitative PCR to ascertain the relative expression level of *LiAG1* in the selected T3-generation transgenic plants, with the objective of identifying those exhibiting a high level of expression for *LiAG1*. The plant height, number of leaves, and flowering time were observed and measured. The morphology of the flowers and floral organs of transgenic *Arabidopsis thaliana* and wild-type *Arabidopsis thaliana* was examined under a microscope.

In order to investigate the effect of the *LiAG1* gene on flower organs of *Arabidopsis Thaliana*, we focused on *AtAP1*, *AtAP2*, *AtSOC1*, *AtFUL*, *AtSEP3*, *AtFT1*, and *AtFT3*, which are related to flower development. We downloaded the above *Arabidopsis* flower development genes from Tair and quantified the expression levels of these genes in three *Arabidopsis* transgenic strains.

### 4.10. Production and Phenotypic Identification of Transgenic Tobacco Lines ‘95’

According to leaf disc transformation [51], the *LiAG1* gene was overexpressed in tobacco, albeit with a few modifications. In short, leaf discs of the WT plants were immersed in the liquid MS medium containing the harvested transformed Agrobacterium cells for about 10 min with occasional shaking. The infected discs were transferred to an antibiotic-free MS medium [MS + 1.0 g/L 6-benzylaminopurine + 0.1 g/L 1-naphthaleneacetic acid (NAA)] and kept in the dark for a co-cultivation period of about 24 h before being transferred to S1 selection medium [MS + 1.0 g/L 6-benzylaminopurine + 0.1 mg/L 1-naphthaleneacetic acid (NAA) + 50 mg/L kanamycin + 400 mg/L hygromycin + 200 mg/L Timentin]. The regenerated buds were transferred to a root-inducing and selection S2 medium [1/2MS + 0.1 g/L NAA + 200 mg/L Timentin]. The selected transgenic tobacco plants were transplanted into the substrate and cultured under the same conditions at 20–22 °C and 16 h/8 h light cycle to obtain tobacco lines with *LiAG1* overexpression.

The DNA of transgenic tobacco plants and wild-type tobacco leaves was extracted using the CTAB method and employed as a DNA template for PCR. The regular expression of the *LiAG1* gene in tobacco plants was identified through reverse transcription polymerase chain reaction (RT-PCR). The overexpression intensity of the *LiAG1* gene in transgenic tobacco lines was determined by semiquantitative RT-PCR, employing specific primers designed with the EF1-α sequence and *LiAG1* gene-specific primers.

The tobacco strains exhibiting the highest *LiAG1* overexpression intensity were selected for comparison with wild-type tobacco with regard to vegetative growth period, flower size, stigma length, and flowering time in the full flowering period.

In order to analyse the expression of genes related to flower organ development in tobacco, *EF1-α* was employed as the internal reference gene. The expression levels of *NtAG*, *NtMADS4*, *NtMADS11*, *NtFT1*, *NtSOC1*, *NtFUL*, and *NtAP1* were analysed in *Nt-OEP3*, *Nt-OEP4*, *Nt-OEP5*, and wild-type tobacco overexpression lines.

### 4.11. Observation of Pollen Viability

In order to verify whether the overexpression of the *LiAG1* gene in tobacco affected pollen viability, the TTC (2,3,5-triphenyltetrazolium chloride) method was used to determine pollen viability. In this method, the pollen is collected at the peak of flowering and placed on a clean slide, and 1 to 2 drops of 0.5% TTC solution is added. Once thoroughly mixed, the glass lid is quickly closed and the slide is placed in a petri dish with a moist filter paper. The petri dish is placed in 37° darkness, and the colouring results are checked after 30 min. Pollen stained red is active, and unstained or lightly stained pollen is inactive [52].

### 4.12. Data Processing and Analysis

All the data in the full text were used for data processing using SPSS 20 software (https://www.ibm.com/support/pages/downloading-ibm-spss-statistics-20 (accessed on 10 June 2023)), data visualisation, and pictures using software such as GraphPad Prism 8.0 (https://www.graphpad.com/updates/prism-802-release-notes (accessed on 10 June 2023)) and Photoshop 2021 (https://helpx.adobe.com/cn/photoshop/using/whats-new/2021-2.html (accessed on 10 June 2023)).

## 5. Conclusions

Finally, this study reports the cloning of the MADS-boxfamily gene *LiAG1* from the Asiatic lily ‘Ice Pink Queen’. It is important to remember that *LiAG1*, which is found in the nucleus, is one of the major C genes influencing stamen formation in the “ABCDE” paradigm. This gene is mostly expressed in the anthers. *LiAG1* silencing will have some effect on the development of pollen production in lily anthers since it will suppress the expression of genes involved in anther development. Overexpression of *LiAG1* impacts the expression of early-blooming genes as well as genes involved in floral organ development, altering the morphology of leaves and floral organs. It also causes early blooming and decreased pollen viability, which affects fertility. *LiAG1* is a critical gene that regulates anther development during floral organ creation, and it may be useful for future breeding of lily variants that lack anthers or pollen.

## Figures and Tables

**Table 1:** The 22 genes identified through NR annotation.

Gene ID	Nr
CL3308.Contig4_All	PREDICTED: GATA transcription factor 19-like
CL14787.Contig1_All	exportin-T isoform X1
CL14787.Contig2_All	FRIGIDA-like protein 4a
CL2611.Contig1_All	FRIGIDA-like protein 4a
CL30.Contig4_All	nodulin homeobox-like isoform X2
CL3768.Contig2_All	agamous-like protein
CL3527.Contig1_All	E3 ubiquitin-protein ligase BRE1-like 2
CL3713.Contig2_All	hypothetical protein COCNU_14G003670
CL5787.Contig2_All	probable histidine kinase 3
CL6866.Contig1_All	FRIGIDA-like protein 4b
CL6885.Contig3_All	flowering-promoting factor 1-like protein 1
CL8146.Contig2_All	pheophorbide a oxygenase, chloroplastic
CL860.Contig2_All	allene oxide cylase
CL8635.Contig3_All	hypothetical protein C4D60_Mb05t18560
CL9067.Contig5_All	FTL1
Unigene26098_All	E3 ubiquitin-protein ligase BRE1-like 2
Unigene30599_All	E3 ubiquitin-protein ligase BRE1-like 2
Unigene37308_All	E3 ubiquitin-protein ligase BRE1-like 2
Unigene42154_All	G-protein coupled receptor 1
Unigene6100_All	phosphatidyl ethanolamine-binding protein
Unigene8761_All	uncharacterised protein LOC103705616 isoform X1
Unigene9076_All	FRIGIDA-like protein 1

## Data Availability

The data presented in this study are available in Supplementary Materials.

## Supplementary Materials

The following supporting information can be downloaded at: https://www.mdpi.com/article/10.3390/plants14030323/s1, Table S1: Statistical of sample analysis results; Table S2: Primer designed in the experiment; Figure S1: Unigene length distribution; Figure S2: CDS length distribution; Figure S3: Group difference volcano map; Figure S4: *MADS-box* gene expression heat map.
