# Supplementary material for: Functional Studies on the LiAG1 Gene of Lilium ‘Ice Pink Queen’ in Flower Development
Source: Plants (Basel). 2025 Jan 22;14(3):323. doi: 10.3390/plants14030323 (PMC11821223; doi:10.3390/plants14030323)

Table S1 Statistical of sample analysis results

Sample	Total Number	Total Length	Mean Length	N50	N70	N90	GC(%)
A1	74072	67740162	914	1492	915	363	45.37
A2	63096	61076282	967	1485	965	421	45.68
A3	64831	62519178	964	1492	962	413	45.57
B1	66363	55132050	830	1280	774	346	45.83
B2	75307	59069809	784	1182	719	329	45.52
B3	70889	55953869	789	1191	719	332	45.73
All-Unigene	148193	140674992	949	1550	951	386	44.99

Table S2 Primer designed in the experiment

Primer name	sequence（5’—3’）
q-CL3786-F	CTCTTATCGTCTTCTCCACCCG
q-CL3786-R	ACGCATCACTGCTTGCTTTCTT
q-Unigene25456-F	AAAAGGTTCTCCAGGAGCACAAG
q-Unigene25456-R	CTCATTTTCGGGACCATTATCTG
q-CL7650-F	GGTGGCAACTTTAGAGCCTTAC
q-CL7650-R	CCATACTCAAAGAACCCAAACC
q-CL16552-F	GAGGTTGCGCTGATCGTCTT
q-CL16552-R	TTTCACCCATCACCTTCCGT
q-Unigene9121-F	TGCCACCTCTTTTGCCTGTA
q-Unigene9121-R	ATCCGTTCCTGTGGTTCTGG
q-Unigene7423-F	CCTTATGTTCTCCAGCACCG
q-Unigene7423-R	TCCAATCCATCCAGCTCCTC
q-CL5970-F	GTTCAGTCAGAAAAGGGAGGTG
q-CL5970-R	TCTGGAATCAAATGTTGGAAGC
q-Unigene34915-F	GAGCTGAAGCGGATCGAGAA
q-Unigene34915-R	CGGCCGTGATTTGAGAAGAT
q-Unigene22517-F	CAGGCAAGAAGCTCCCATACAC
q-Unigene22517-R	TGGCTTGAAATAAACCCTCTGA
q-Unigene12340-F	CCTTGGCGAGAACTTGGGTC
q-Unigene12340-R	CTGGCTTCCTCCAACTGCTT
q-CL15387-F	GAGGGAGATAAGGCGGATTG
q-CL15387-R	CAGCTGGAGAACTCGCAGAG
q-Unigene7613-F	CCTTATGTTCTCCAGCACCG
q-Unigene7613-R	TCCAATCCATCCAGCTCCTC
LiAG1-F	ATGGGTAGGGGTAAGATAGAGATCAAG
LiAG1-R	TTAACCTAGTTGGAGGGCAGTCTGC
egLiAG1-F	agctcggtacccggggatccATGGGTAGGGGTAAGATAGAGAT
egLiAG1-R	accccgctcaccatgtcgacACCTAGTTGGAGGGCAGTCT
TRV-LiAG1-F	agaaggcctccatggggatccGAAAACGAGAGATCACAGCAGCA

（Carried down）

（Brought forward）

Primer name	sequence（5’—3’）
TRV-LiAG1-R	tgtcttcgggacatgcccgggATGGCTTCGAAAGCACTTGTG
q-TIP41-F	GTTTATTCTTCTCCGTTTCTGGC
q-TIP41-R	GGTTTGGCTTTTGGGTCGTT
q-LiAG1-F	CTGCTATTGAGGCGGGAAAA
q-LiAG1-R	TGGCAAGCCCTGGATACACT
q-LiSEP3-F	GTATGTTGGGTGTGTGGGGAAT
q-LiSEP3-R	GCAGTCCATTACAACACTCCTG
q-LiSEP2-F	ATTCAACGGCTTGGCATCG
q-LiSEP2-R	TCACGTCGTCGGTAAGGGAGTC
q-LiSEP1-F	GGTGCGACGGTAGACGATTG
q-LiSEP1-R	CCGGAAGTGCTTCAACGAA
q-LiAP2-F	GGGACAAGACATTACAAAGGAGACT
q-LiAP2-R	TCCCTTGCGACTTCAGTAGAGC
q-LiWUS-F	CATTGCTCGATCAGCACATT
q-LiWUS-R	GAGCAATGCGACCGAATTTG
q-AtActin-F	CTCCCGCTATGTATGTCGCC
q-AtActin-R	GTCACGTCCAGCAAGGTCAAG
q-NtActin-F	ACGCAAGTACAGTGTCTGGA
q-NtActin-R	AAGCATTTGCGGTGGACAAT
q-AtAG-F	TTGGGAAGCTCGAATGGG
q-AtAG-R	AGCATACAAAACTCCAACAGGC
q-AtAP1-F	TCCGAACTGTCTCAGCCTTG
q-AtAP1-R	CAGGCAAGAAGCTCCCATACAC
q-AtAP2-F	ACTGTCAGGGTTTGGGATGC
q-AtAP2-R	GGGATGGAAGTCAAGTGATGTAA
q-AtSOC1-F	CCTGCCAACATTTCAGTCTCC

（Carried down）

(Brought forward)

Primer name	sequence (5'—3')
q-AtSOC1-R	GACTCGTTTCCATCAACCTCAC
q-AtFUL-F	ACCAAGGTGACGAACCAAGTATT
q-AtFUL-R	AGTGAACGTAGTGTCGCATTTTAG
q-AtFT1-F	TGTACGAGTTTTGCAGTAGTTCGA
q-AtFT1-R	GGGTTCTGGTGCTCCATAGTT
q-NtAG-F	GCAACCCTAACCTGAGGGAG
q-NtAG-R	TACACAGTTTCGCGACCCAA
q-NtMADS4-F	GCAGCAGCAACTGCTAACAATA
q-NtMADS4-R	TAGGATACAGATGATTTTGGCGT
q-NtMADS11-F	TGCTGAGAGGCAGCTTACTG
q-NtMADS11-R	TGCAAAACCTCAAATCTTGCCT
q-NtSOC1-F	GGTGGCCTTCAACAGAGACA
q-NtSOC1-R	GGGACGCCTTATTCTGCACT
q-NtFT1-F	ACAAGTCTACAATGGTGGTGGT
q-NtFT1-R	GTGAAGGTCGTGGACTCTCG
q-NtFT3-F	TGCTGTTTCGACAATTGACTC
q-NtFT3-R	GTACATAGCAGCAACAGGCG
q-NtFUL-F	CCTTACACATTTTCTCAGACCAA
q-NtFUL-R	ATGTGCTTTCTTCGCTAAACCTC
q-NtAP1-F	GCTAATACTGTGATGCCCCCA
q-NtAP1-R	GTTATTCAAAGGCAAGGCTGAT

Note: The small and medium letters of the primer are the homologous arm sequence of the carrier, "q-" is the fluorescent quantitative PCR primer, and the annealing temperature of the primer conforms to the application specification.

Figure S1 Unigene length distribution

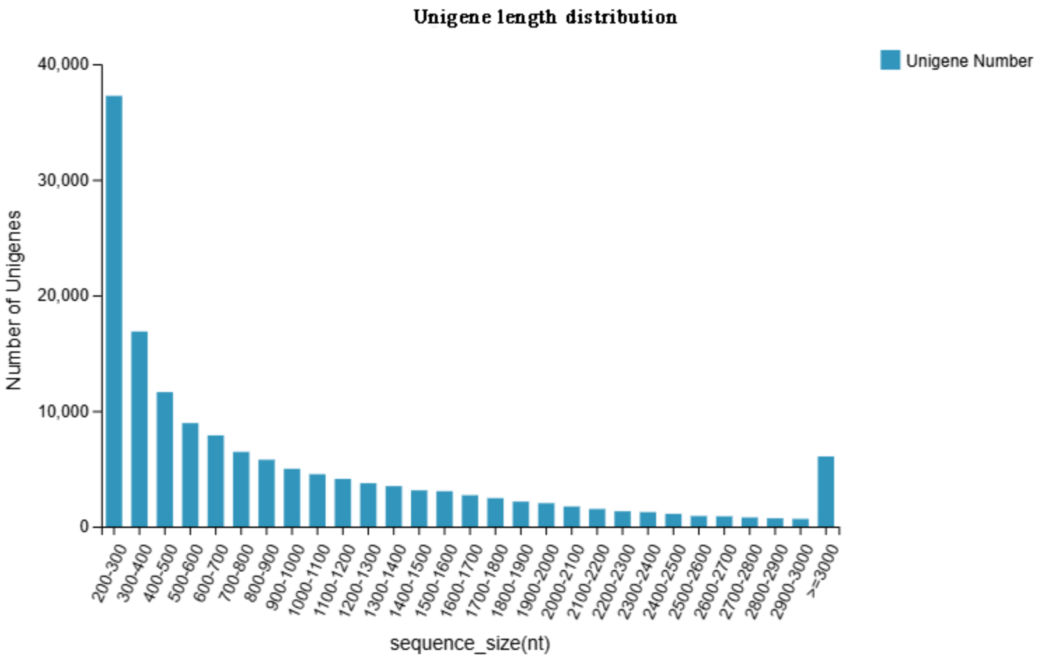

Figure S2 CDS length distribution

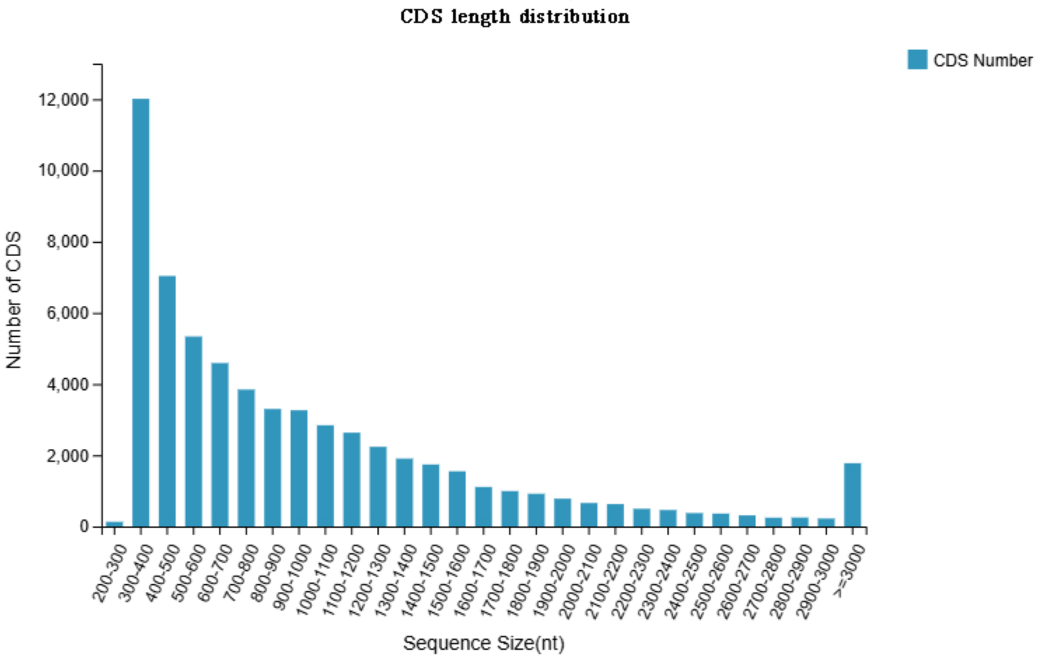

Figure S3 Group difference volcano map

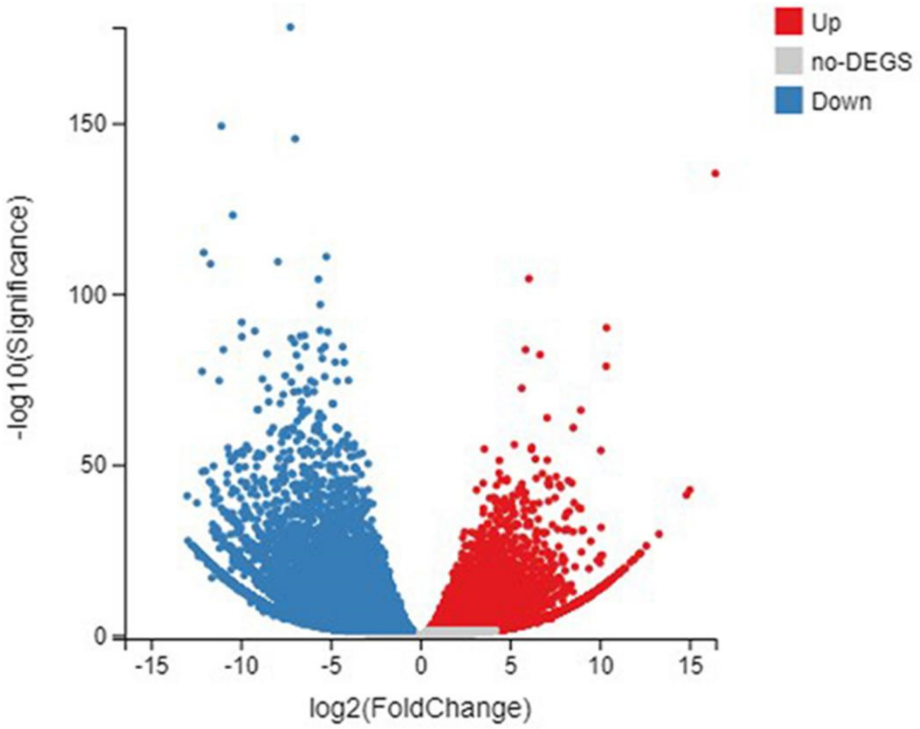

Figure S4 *MADS-box* gene expression heat map

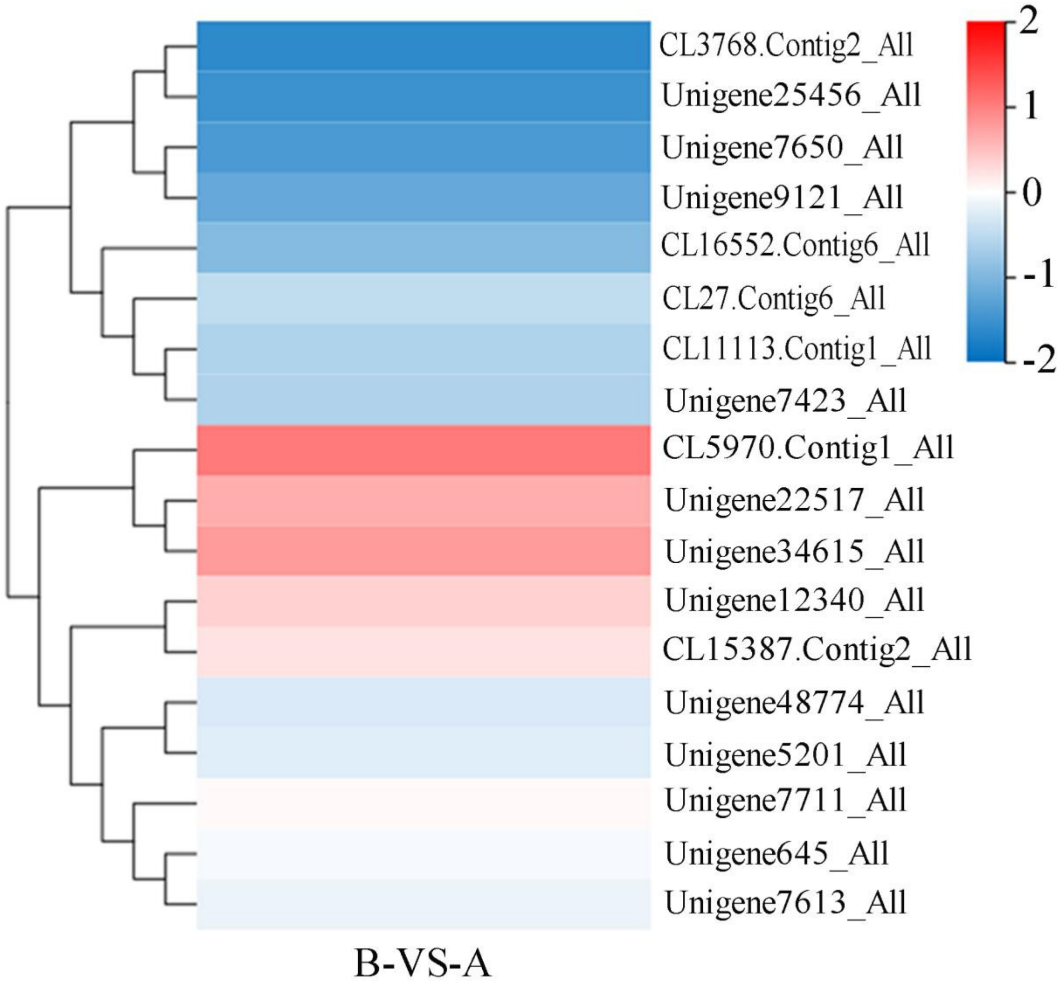

Supplement: Supplementary file 1 [file plants-14-00323-s001.zip › plants-3283505-supplementary.pdf]
